# Use of acoustic emission to identify novel candidate biomarkers for knee osteoarthritis (OA)

**DOI:** 10.1371/journal.pone.0223711

**Published:** 2019-10-16

**Authors:** Daniela K. Schlüter, Lucy Spain, Wei Quan, Harry Southworth, Nicola Platt, Joe Mercer, Lik-Kwan Shark, John C. Waterton, Mike Bowes, Peter J. Diggle, Mandy Dixon, Jane Huddleston, John Goodacre

**Affiliations:** 1 CHICAS, Lancaster Medical School, Lancaster University, Lancaster, England, United Kingdom; 2 Institute of Population Health Science, Liverpool University, Liverpool, England, United Kingdom; 3 Faculty of Health and Medicine, Lancaster University, Lancaster, England, United Kingdom; 4 Institute of Science, Natural Resources and Outdoor Studies, University of Cumbria, Carlisle, England, United Kingdom; 5 Applied Digital Signal and Image Processing Research Centre, University of Central Lancashire, Preston, England, United Kingdom; 6 Data Clarity Consulting Ltd, Altrincham, England, United Kingdom; 7 Lancaster Health Hub, Lancaster University, Lancaster, England, United Kingdom; 8 The Christie NHS Foundation Trust, Manchester, England, United Kingdom; 9 Centre for Imaging Sciences, University of Manchester, Manchester Academic Health Sciences Centre, Manchester, England, United Kingdom; 10 Imorphics Ltd, Manchester, England, United Kingdom; Toronto Rehabilitation Institute - UHN, CANADA

## Abstract

Our objective was to determine the efficacy and feasibility of a new approach for identifying candidate biomarkers for knee osteoarthritis (OA), based on selecting promising candidates from a range of high-frequency acoustic emission (AE) measurements generated during weight-bearing knee movement. Candidate AE biomarkers identified by this approach could then be validated in larger studies for use in future clinical trials and stratified medicine applications for this common health condition. A population cohort of participants with knee pain and a Kellgren-Lawrence (KL) score between 1-4 were recruited from local NHS primary and secondary care sites. Focusing on participants’ self-identified worse knee, and using our established movement protocol, sources of variation in AE measurement and associations of AE markers with other markers were explored. Using this approach we identified 4 initial candidate AE biomarkers, of which “number of hits” showed the best reproducibility, in terms of within-session, day to day, week to week, between-practitioner, and between-machine variation, at 2 different machine upper frequency settings. “Number of hits” was higher in knees with KL scores of 2 than in KL1, and also showed significant associations with pain in the contralateral knee, and with body weight. “Hits” occurred predominantly in 2 of 4 defined movement quadrants. The protocol was feasible and acceptable to all participants and professionals involved. This study demonstrates how AE measurement during simple sit-stand-sit movements can be used to generate novel candidate knee OA biomarkers. AE measurements probably reflect a composite of structural changes and joint loading factors. Refinement of the method and increasing understanding of factors contributing to AE will enable this approach to be used to generate further candidate biomarkers for validation and potential use in clinical trials.

## Introduction

Knee osteoarthritis (OA) is a common degenerative joint condition, particularly amongst older people. The diagnosis of knee OA relies on X-radiology and involves a combination of structural features and pain symptoms [1]. However, X-ray features correlate poorly with pain symptoms and are not useful in diagnosing early knee OA [2]. Whereas the research and management of other health conditions has been advanced by identification of biomarkers [3] the paucity of biomarkers limits the potential for clinical trials to evaluate new treatments for knee OA. Furthermore, the paucity of biomarkers limits the extent to which principles of stratified medicine might be applied to knee OA, particularly given the clinical and biological heterogeneity of this condition. However, the recent development of techniques to measure high-frequency acoustic emission (AE) from knees now offers the possibility of identifying AE features which reflect the integrity of interactions between joint components during weight bearing movement [4–9]. Such features would be regarded as "biomarkers" in the sense adopted by the NIH/FDA BEST resource [3], where molecular, histologic, radiographic, or physiologic characteristics (including, potentially, AE signatures) are types of biomarker. This approach has face validity with a clear rationale for enabling the identification and development of new knee OA biomarkers.

AE-based techniques are well established in non-destructive testing and condition monitoring of engineering structures, enabling early detection of damage and defects. By analogy, smooth and well-lubricated surfaces move quietly against each other, whereas uneven movements of rough, poorly-lubricated surfaces generate acoustic signals.

Exploration of the use of AE in health and medical applications is as yet at an early stage, and the origins of acoustic signals in joints and other body structures is as yet unknown. However, knee OA is known to involve damage and defects in cartilage and bone, and by analogy the impact of these changes on the articulation and dynamics of moving joint surfaces under weight bearing conditions is likely to be a major basis for generating the acoustic signals captured by this method. The fact that damage to cartilage and bone are prominent structural features of knee OA, and that these features may worsen over time, provides us with a rationale for investigating the use of AE-based biomarkers, since in principle these would reflect directly the key pathological feature of this condition.

Previously we have developed a non-invasive, portable system and a standard protocol for measuring high-frequency AE in knees during weight-bearing movement to identify differences in AE from healthy and OA knees in different age groups [4–9]. We are now exploring whether AE may have applications in clinical trials for people previously-diagnosed with knee OA.

The aim of this study was to determine whether AE measurements from knees of people with previously-diagnosed knee OA can be used to identify novel candidate biomarkers. Potentially, any AE candidate biomarkers identified using this approach could then be validated in larger studies for use in future clinical trials and stratified medicine applications for this common health condition. Using a population cohort approach we focused on two key issues, both of which reflect fundamentally important criteria for potential new biomarkers for knee OA. Firstly, are AE measurements capable of distinguishing between different grades of knee OA severity, and secondly are AE measurements sufficiently reproducible to have potential utility as biomarkers? Our results show that AE measurements have clear potential to fulfil both these criteria and demonstrate the feasibility of identifying new candidate biomarkers for knee OA, based on AE measurements, by developing further this novel approach.

## Methods

### Study design

89 adults with X-ray Kellgren-Lawrence (KL) scores of 1 or higher were recruited from NHS primary and secondary care sites across Lancashire and South Cumbria. People with significant co-morbidity or previous knee surgery were excluded. All participants were invited to local GP or hospital clinics for AE measurement and clinical assessment, either for 1 day or for repeated measurements on 3 consecutive days and 3 consecutive weeks. For those who consented to repeated measurements (n = 45), each participant underwent AE measurement by each of 3 different AE-trained NHS research practitioners (RPs) on day 1. After completing AE measurement, the sensor was removed, then re-attached by the next RP. Measurements on subsequent days were made by the same RP. All measurements were taken in NHS clinic environments. All participants undertook only normal daily weight-bearing activities prior to measurements.

Joint angle and acoustic emissions were simultaneously recorded from both knees using a ‘Joint Acoustic Analysis System’ (JAAS), comprising an AE sensor and electro-goniometer connected to a computer. Electro-goniometers were positioned laterally to each knee, along the plane between greater trochanter and lateral malleolus. AE measurements were made simultaneously on the worse knee (as identified by the participant, and for which an X-ray had been taken within the previous 4 weeks) and the other knee, using one of 3 JAAS machines. Radiographs were acquired during standard care at participating NHS Trusts and analysed centrally. KL scores for the worst knee were assessed and agreed independently by 2 experienced musculoskeletal radiologists.

### Acoustic emission

Wide-band AE S9204 sensors (Mistras Group Ltd), with a high sensitivity in the range of 50kHz– 200kHz recorded bursts of acoustic energy generated by stress and friction between joint components during weight-bearing movement. AE data acquisition operates in event-based recording mode, such that signals must have significant amplitude to be recorded as an AE “hit”. Sensors were positioned on both knees anterior to the medial patella retinaculum, and a thin covering of vaseline applied to ensure good acoustic coupling. Based on previous work, machine threshold setting for data acquisition was 36 dB, with respect to the 1 μV level, using a frequency range of 20 to 400 kHz and 5 MSPS sample rate. Data from a concurrent study involving 73 other participants, with data collected by the same RPs using the same protocol but with machines calibrated at 20 to 80 kHz, and using a 1 MSPS sample rate to enable recording of longer waveforms, were used to support interpretation of reproducibility and KL correlation data.

### AE data collection

RPs received detailed training in equipment and data collection protocols, and were supported closely by the study team to ensure standardized protocol use. AE and joint angle were recorded simultaneously from both knees whilst participants performed sit-stand-sit movements, starting in a seated position with their back against the chair and knees bent at 90 degrees. Each test involved 2 sets of 5 sit-stand-sit movements, following an initial practice of 5 movements with sensors attached. Participants were asked to perform movements as smoothly as possible at a speed comfortable for them.

### Clinical data collection

RPs collected demographic and clinical data at the first assessment, including age, sex, BMI, weight, pain in the worst knee visual analog scale (VAS), pain in other knee (yes/no), and pain, function and stiffness scores using WOMAC. Data were recorded in a purpose-designed sheet, together with the KL score for the X-ray of the worst knee.

### MR imaging and segmentation

29 participants volunteered to also undergo MR imaging of the worst knee within 4 weeks of AE and clinical assessment. MR images were acquired with a 3.0T Philips Achieva-X using a Philips 16-element SENSE knee coil [10]. Sagittal fat-saturated MR images were acquired using a Philips 3D WATSf sequence. Bone and cartilage were segmented by an experienced manual segmenter, using a semi-automated livewire algorithm (Endpoint segmentation software, Imorphics, Manchester, UK). Bone surfaces were automatically segmented using active appearance models (AAMs), built from an independent training set. AAMs were applied to the same 3D WATSf sequences [11]. Manual segmented bone surfaces were used for comparisons across the population. Cartilage thickness measures were taken using correspondence points on bone surfaces (S1 Text, S2 Text).

### Data analysis: Identifying initial candidate AE biomarkers

A developmental dataset comprising 8 participants was derived from the cohort. Of these, 5 had “clinically-mild” knee OA (KL score of 1 and mild or intermittent pain with little or no functional impairment) whilst 3 had “clinically-severe” knee OA (KL score of 3 or 4 with significant and persistent pain, and functional impairment). Initial candidate AE biomarkers which segregated clearly with clinical severity within the developmental dataset were identified, of which 4 candidates were selected for detailed analysis using data from the remaining cohort. Whilst one of the candidates was directly derived from the number of AE hits above the 36 dB threshold setting of the machine, the other three candidates were derived based on the methodology reported in [8]. It consisted of processing joint angle signals to divide each sit-stand-sit movement into 4 quadrants, extracting average signal levels and peak signal amplitudes from AE hits in each movement quadrant, forming the AE feature profile of each knee in the developmental dataset based on two-dimensional histograms, and applying Principal Component Analysis (PCA) to yield the first three principal components as the three candidates.

### Data analysis: Evaluating initial candidate AE biomarkers

The 4 initial candidate biomarkers were evaluated with respect to reproducibility and their associations with participant characteristics and disease specific markers. To assess variability between patients, between machines, between RPs and between visits, and to account for variability introduced by the application of sensors, which is not necessarily a systematic difference between RPs, 2 separate linear mixed effects models were fitted; a ‘day one’ model and a longitudinal model. Both models included a fixed effect term for JAAS machines and random effects terms to capture between-patient variability, between- RP variability and residual error. The longitudinal model further included a random effect capturing day-to-day variability whereas the ‘day one’ model included a random effect for between-session variability within a day. A 'session' is a time unit during which the sensors are not removed and re-applied. The models were fitted separately for each of the candidate biomarkers. The models were developed using data from 45 participants who consented to take part in the repeated measurements study.

To assess associations with other markers and patient characteristics, a linear mixed effects model was fitted for each candidate biomarker. The model included fixed effects for covariates of interest, a participant- specific random effect that accounts for correlation between repeated measurements within the same individual, and a residual error term. A multiple regression model was developed using forward selection based on the likelihood ratio test with a cut-off for significance of p<0.1. The model was developed on 68 participants who had complete data and the final model was refitted using data from 76 participants for whom complete data were available on the covariates used in the final model. Data used in the developmental dataset were excluded.

All analyses were performed using the statistical computing environment R and the `lme4' package. Parameters in final models were estimated using restricted maximum likelihood. Confidence intervals provided are based on the likelihood profile (S3 Text).

### Ethics statement

The study was reviewed and approved by Health Research Authority National Research Ethics Service Committee North West–Lancaster Research Ethics Committee reference 13/NW/0732.

Written consent was gained by all participants and a signed copy held in the site file.

## Results

**Fig 1:**
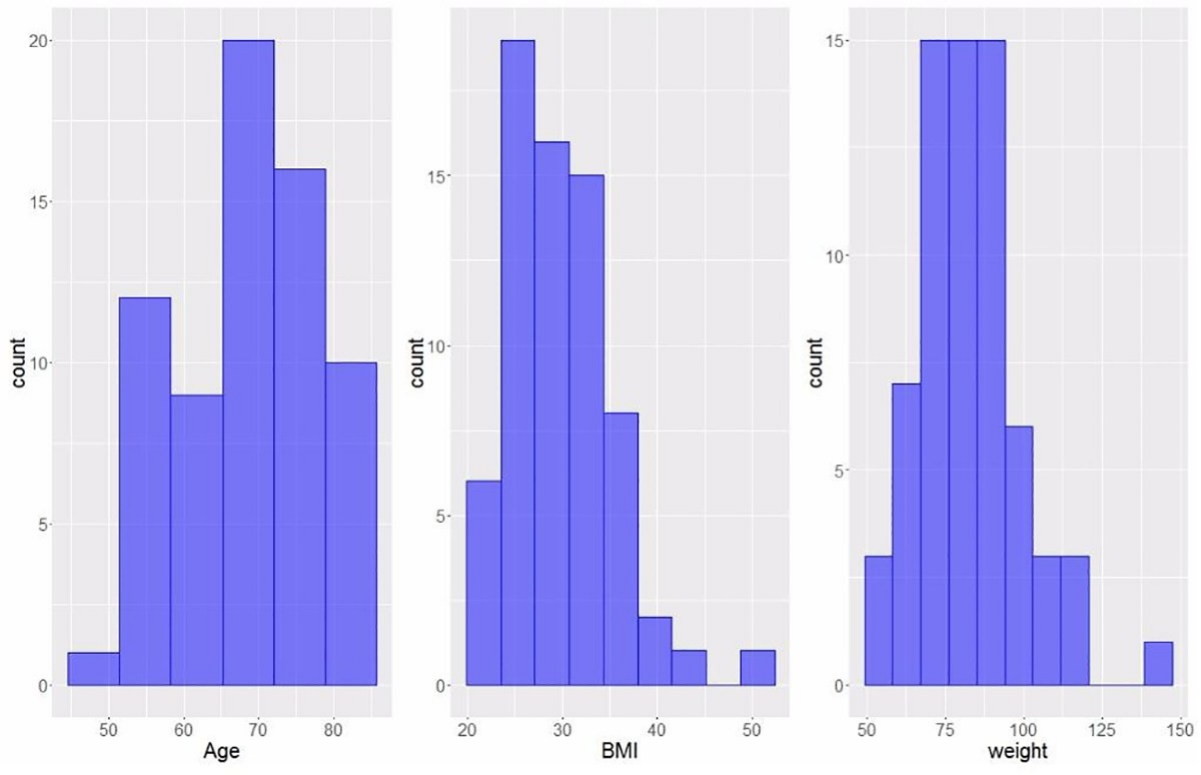
Distribution of age, BMI and weight profiles of participants (n = 68) with complete datasets. Within this group, 12 participants had a KL1 score, 22 had KL2, 27 had KL3 and 7 had KL4.

Age, BMI and weight profiles of participants for whom complete data sets were available (n = 68, comprising 36 males, 32 females) are shown in Fig 1, and WOMAC scores for Pain, Stiffness and Function in this group are shown in Table 1.

**Table 1:** WOMAC score profile of participants (n = 68). The table shows median and IQR values for each domain of Pain, Stiffness and Function.

WOMAC scores	Median	interquartile range
Pain	17	6–28
Stiffness	10	4–13
Function	61.5	16–90

**Fig 2:**
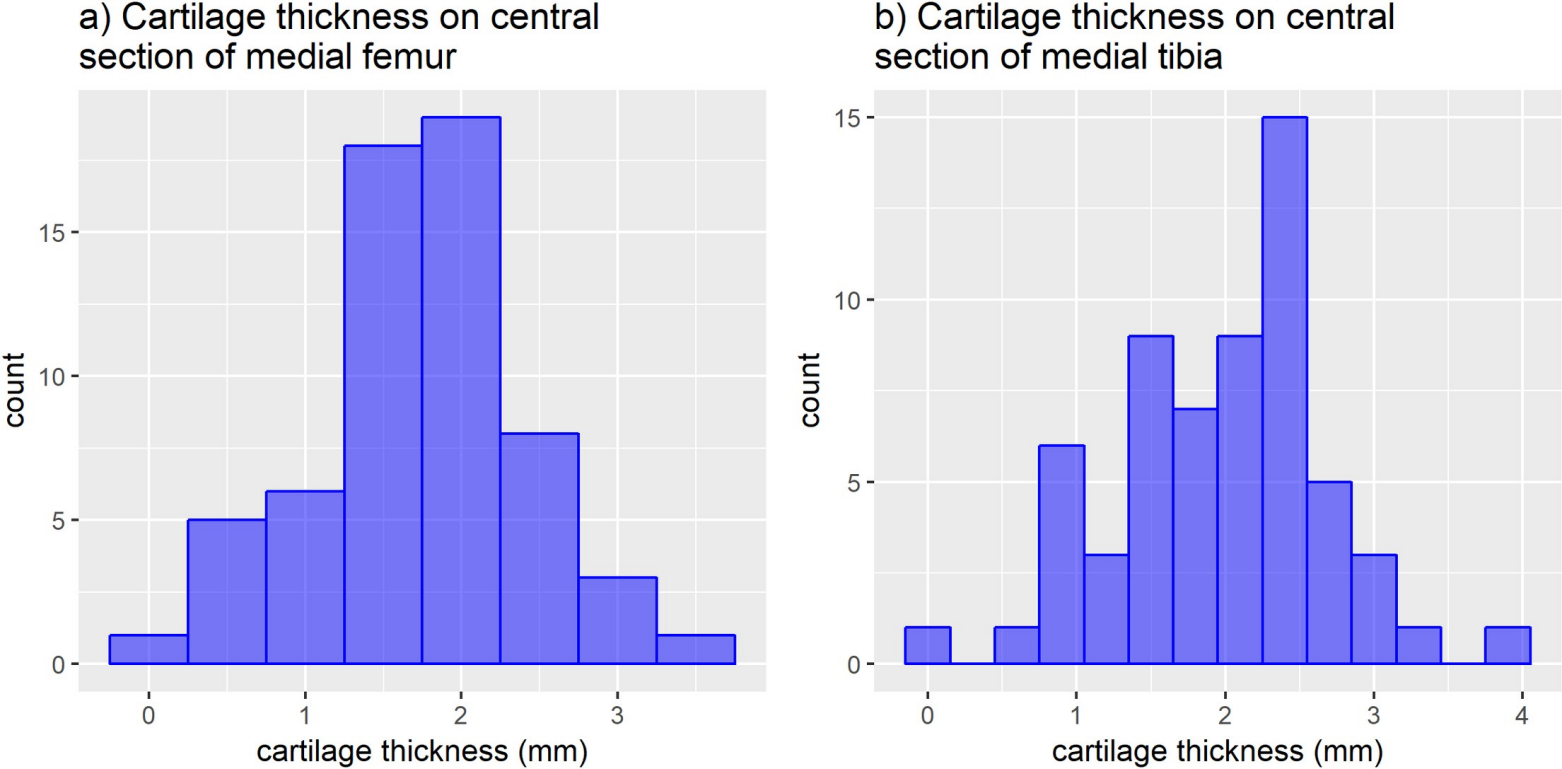
Distribution of cartilage thickness (n = 29). a) Thickness of cartilage on the central medial femoral condyle (anterior aspect) over total area of subchondral bone representing subchondral bone area. Peripheral osteophytes are excluded, base of central osteophytes are included. b) Thickness of cartilage on the central medial tibia over total area of subchondral bone representing premorbid subchondral bone area. Peripheral osteophytes are excluded, base of central osteophytes are included.

Fig 2 shows the distribution of central femoral condyle and central medial tibia cartilage thickness measurements among the 29 participants who underwent MRI studies.

### Acceptability and reproducibility of AE measurements

The protocol was feasible and acceptable to all participants and RPs. Although a few participants reported knee discomfort after the test, all were able to complete the protocol, and no significant difficulties were observed or reported regarding symptoms or mobility. This is consistent with our previous work, and supports the potential for using this technique in multicentre clinical trials.

**Fig 3:**
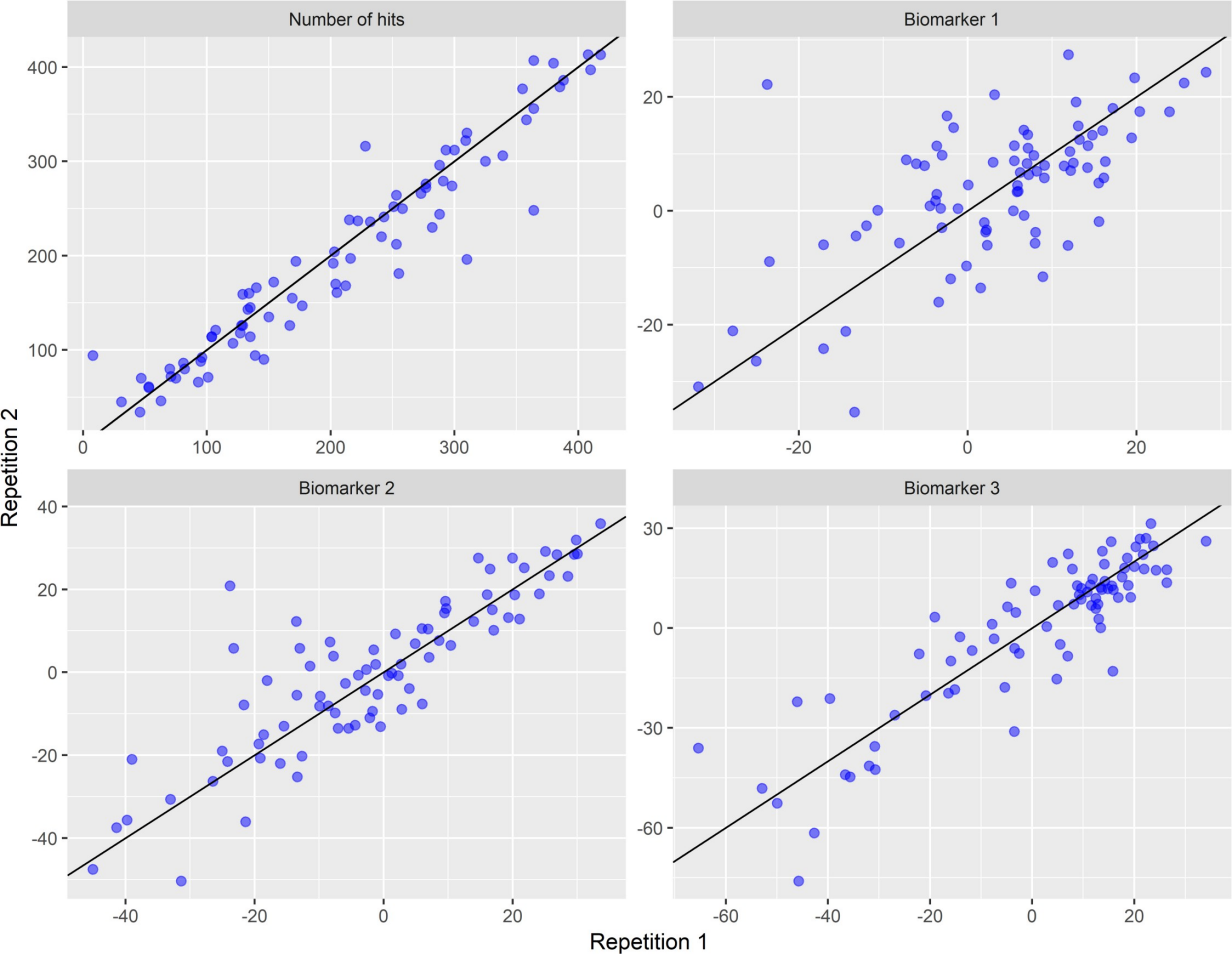
Within-session repeatability of each AE candidate biomarker. Each graph shows the biomarker value obtained from the first set of sit-stand-sit movements (x-axis) plotted against the biomarker value obtained from the repeat set of movements (y-axis). Number of hits contained one outlier at (254, 873). This measurement was omitted from the figure. S1 Fig shows the outlier included.

Reproducibility was assessed in terms of within- session, day to day, week to week, between-practitioner, and between- machine variation. Repeatability was good for all 4 candidates when comparing the first and second sets of 5 movements within a session (Fig 3). “Number of hits” showed strongest within-session repeatability, and the standard deviation (SD) of measurements within the same session within the same patient was estimated to be 18.74—approximately half the SD of the “number of hits” measured in different sessions (46.02) and a quarter of the SD of measurements taken on different participants (82.06; Table 2, Part A). Table 2, Part B shows estimated contributions to variability for “number of hits” from different sources in the longitudinal model. Variability between participants was higher than variability due to day of measurement, RP and JAAS machine. However, the 3 PCA-based candidates showed relatively low levels of variability between participants, and also suggested a possible effect of JAAS machine on the measurement.

**Table 2:** Variability profile for AE “number of hits”. **Table 2**: **Part A**: Point estimates and 95% confidence intervals for the standard deviations of the random effect terms and the regression coefficients of the JAAS machine relative to JAAS 1 in the ‘day 1’ model of the ‘Number of hits’. (LCL: lower confidence limit; UCL: upper confidence limit). **Table 2: Part B**: Point estimates and 95% confidence intervals for the standard deviations of the random effect terms and the regression coefficients of the JAAS machine relative to JAAS 1 in the longitudinal model of the ‘Number of hits’. (LCL: lower confidence limit; UCL: upper confidence limit).

Parameter	Model without covariate adjustment			Model with covariate adjustment		
	Point Estimate	95% LCL	95% UCL	Point Estimate	95% LCL	95% UCL
**A**						
Session in patient variability	46.02	38.84	55.12	46.56	39.25	55.81
Patient variability	82.06	62.65	101.12	79.11	51.96	86.62
RP variability	6.05	0.00	22.65	5.77	0.00	22.50
Residual variability	18.74	16.60	21.37	18.58	16.44	21.22
JAAS 2	20.39	-36.87	77.61	19.57	-36.70	75.97
JAAS 3	-66.38	-137.07	4.17	-52.83	-126.68	21.03
**B**						
Day in patient variability	55.75	49.71	62.93	55.98	49.47	63.39
Patient variability	79.15	60.55	101.40	74.49	48.04	87.16
RP variability	47.22	0.00	87.92	47.72	0.00	94.14
Residual variability	19.42	17.63	21.54	19.85	17.95	22.11
JAAS 2	1.16	-68.49	67.53	1.33	-68.37	69.58
JAAS 3	-26.77	-117.85	59.25	-4.08	-113.57	99.76

Similar results were found in a concurrent study involving 73 other participants, with machines calibrated at a narrower frequency range of 20 kHz to 80 kHz. Although the narrower frequency range generated different AE outputs, it did not lead to differences in data reproducibility or in relative contributions to measurement variability from the different factors investigated (S1 Table).

These results show that differences between participants contributed the largest source of variation in “number of hits”, supporting the rationale for focusing on “number of hits” measurement for potential use in clinical trials.

### Associations between initial candidate AE biomarkers and other markers

Whilst AE profiles in the worst knee differed from those in the contralateral knee for all 4 candidates, “number of hits” also showed significant associations with disease or other markers. Therefore, for further analysis we focused only on “number of hits” for its potential as a candidate biomarker.

**Fig 4:**
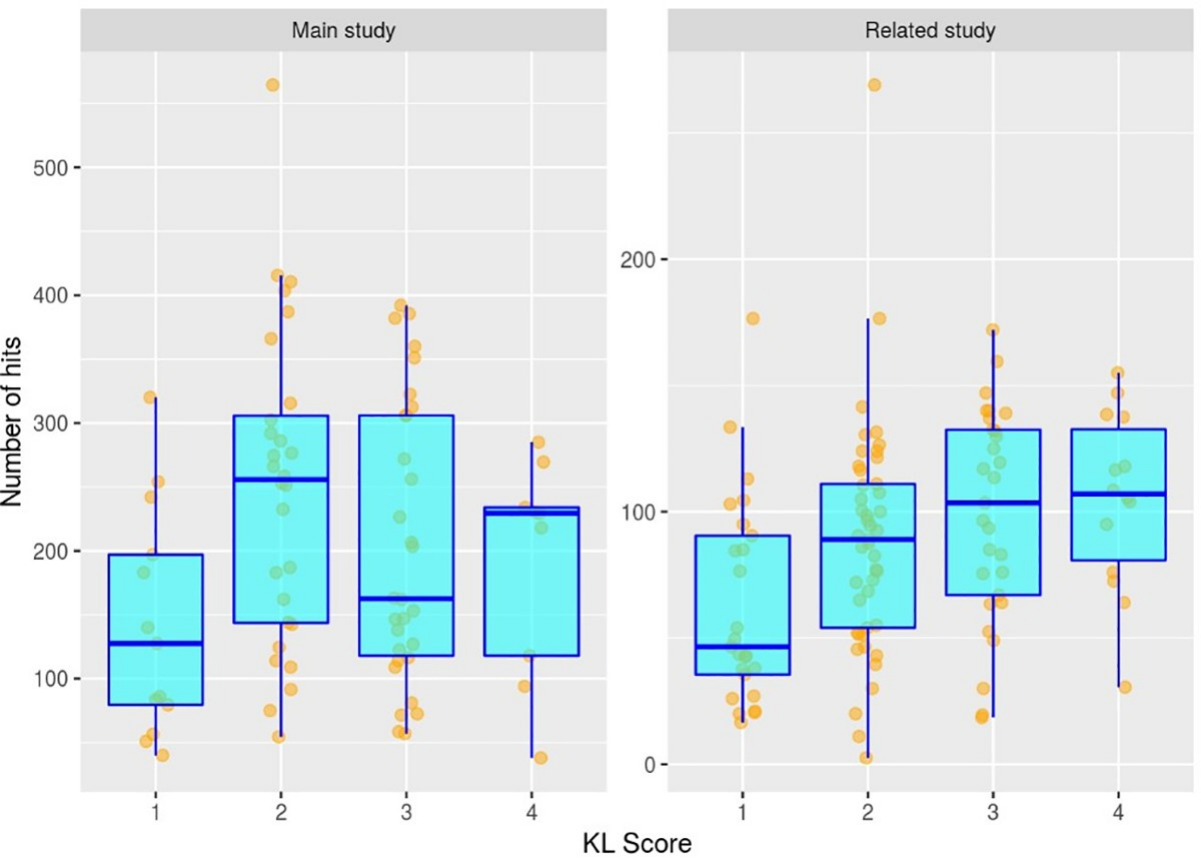
Relationship between AE number of hits and KL score. Fig 4 left panel shows the relationship between AE number of hits and KL score for the main study group. Fig 4 right panel shows the relationship between AE number of hits and KL score for the concurrent study group (n = 73) for which data were collected using a frequency range of 20 kHz—80 kHz.

“Number of hits” was lower in knees with KL score 1 compared with KL score 2, but did not distinguish between KL scores 2 and 3, or 3 and 4 (Fig 4, Table 3). The same result was obtained in the analysis of the other dataset. However, there were qualitative differences between the AE outputs from the two datasets (Fig 4). Although there was no clear trend in the number of hits by KL score in our main study (see also S2 Table), a trend was apparent in the other dataset. This raises the possibility that machine frequency settings may have a role in the identification of further AE candidate biomarkers. Interestingly, whilst “number of hits” was not associated with pain in the ipsilateral knee, it was significantly associated with pain in the contralateral knee and with body weight (Table 3). S3 Table shows results from the univariable analyses.

**Table 3:** Parameter estimates in multiple regression model for AE number of hits. Point estimates and 95% confidence intervals for parameter estimates in the multiple regression model for the ‘Number of hits’. (LCL: lower confidence limit; UCL: upper confidence limit). The intercept is the average number of hits across all KL scores in individuals of mean weight and no pain in the contralateral knee.

Parameter	Point Estimate	95% LCL	95% UCL
Intercept	160.13	124.67	195.6
KL 1 vs KL 2	-81.77	-143.41	-20.13
KL 2 vs KL 3	22.54	-25.87	70.95
KL 3 vs KL 4	38.39	-33	109.78
Weight	2.06	0.76	3.37
Pain in contralateral knee	57.05	14.13	99.97
Standard deviation of participant specific random effect	93.17	76.58	105.98
Standard deviation of residual error	22.51	19.39	26.57

There was no evidence of significant relationships between “number of hits” and any of the measures of cartilage thickness used in this study, involving either medial femoral central or medial tibial central cartilage (S3 Table).

### Analysis by movement quadrant

**Fig 5:**
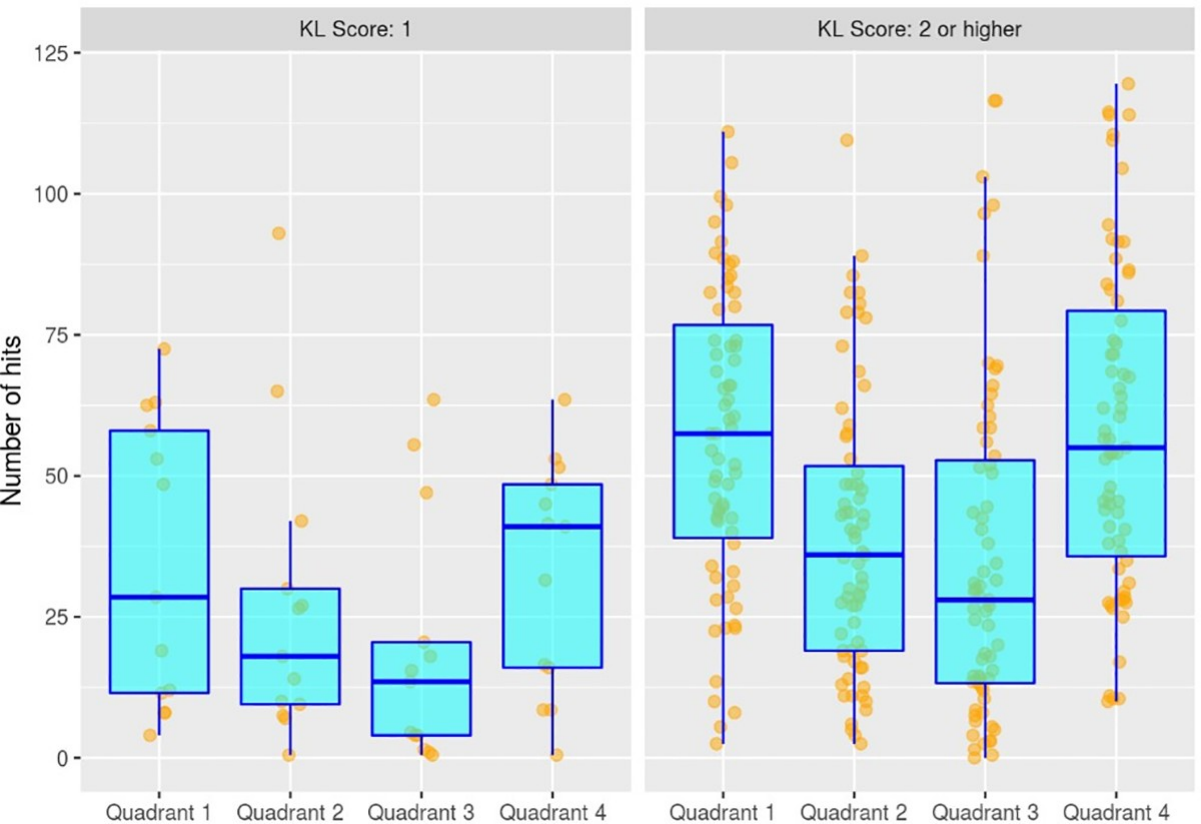
Relationship between AE number of hits and movement quadrant. The distribution of AE number of hits by each movement quadrant is shown for participants with KL scores of 1 and KL scores of 2 or higher.

By dividing movements into 4 quadrants corresponding to different phases of ascending / descending and accelerating / decelerating movement, we found that "number of hits" was consistently higher in movement quadrants 1 and 4, corresponding to phases of “ascending-acceleration” and “descending-deceleration” respectively, across all OA severity groups (Fig 5). Furthermore, the effect of "pain in the contralateral knee" on "number of hits" was strongest in the descending-deceleration phase, suggesting that AE due to factors in the other knee may also be quadrant-related.

## Discussion

There is increasing interest in potential applications of AE in musculoskeletal and other clinical conditions [12–22], and it has been demonstrated that AE is associated with experimental cartilage damage in an in vitro equine model [23]. The recent development of techniques for analysing AE extends previous concepts using vibration signals to inform diagnosis of knee conditions [24]. For example, Lee et al [25] used vibration arthrometry to identify OA subgroups among 36 people with patella femoral crepitus, based on location of cartilage damage, whilst Krishnan et al [26] investigated vibroarthrography in diagnosing chondromalacia patellae by studying 39 people with knee conditions and 51 people with normal knees. However, although vibroarthrography and AE share some similarities conceptually, the principles and protocols involved differ markedly. In particular, our AE technique focuses on capture and analysis of high frequency acoustic signals (20 to 400 kHz) emitted from knees during weight bearing movement. We have developed a convenient, non-invasive and portable system to capture and analyse high-frequency sound emitted during weight-bearing knee movement [4–9] and are focusing on AE as a marker of knee OA severity, rather than as a diagnostic tool. AE reflects aspects of joint function to which conventional imaging and soluble biomarkers are insensitive. It reflects dynamic interaction among joint components as a result of movement, and complements traditional imaging biomarkers which reflect static anatomy. Unlike many existing clinical measures, AE offers an objective and quantitative measurement tool, which can be standardized across centres and patients.

In this study we investigated whether AE measurements can be used to identify novel candidate biomarkers for potential use in clinical trials and stratified medicine applications. Given the heterogeneity of knee OA, the availability of biomarkers to enable better definition of patient subgroups and better, more objective assessment of the outcome of interventions would support the design of multicenter clinical trials for this condition. Our aim was to determine the efficacy and feasibility of a new approach for identifying candidate biomarkers for knee OA, based on a strategy of selecting promising candidates from a range of acoustic emission (AE) measurements generated during weight-bearing knee movement. Our rationale was that if it proved possible to identify candidate biomarkers by screening AE data generated using a defined knee movement protocol, such candidates could then be further tested and validated in larger scale studies. Furthermore, the same principles and approach could be used in future studies to generate further candidates, perhaps opening the way for characterising patient subgroups, and enabling stratified medicine approaches to be explored for knee OA. In this initial study we focused in particular on the acceptability and reproducibility of an initial set of selected AE candidate biomarkers, and on their associations with other knee OA markers. This study is the first to investigate AE in a large population cohort with previously-diagnosed knee OA, and also the first to address systematically the reproducibility of AE measurement. Although this field is as yet at an early stage, our findings demonstrate the potential for using this approach in biomarker generation and clinical trials, as well as identifying areas for further technical development and improvement.

Participants with a range of knee OA severity were measured by trained RPs in primary care and secondary care settings. Use of the equipment or the protocol caused no significant problems necessitating discontinuation of the test. We anticipated that the protocol might prove challenging for some participants to complete (S2 Fig) but this proved not to be the case Furthermore, none of the RPs reported significant practical difficulties in using the JAAS, although a rigorous training programme and access to technical advice throughout data collection were key factors in ensuring acceptability of the technique.

We initially identified 4 candidate AE biomarkers for detailed analysis. Of these, “number of hits” showed strongest potential for further development as a candidate biomarker. The “number of hits” measurement showed higher levels of inter-participant variability compared to other sources of variability, and there was a difference on average in “number of hits” between knees scoring KL1 or KL 2. The same results were obtained from another dataset collected using the same equipment and protocol but with a narrower frequency range. This dataset showed a trend of increasing number of hits with increasing KL score. Although differences between KL2 and KL3, and KL3 and KL4 were not statistically significant, this suggests that machine frequency settings may have a role to play in the further development of AE biomarkers. No associations were found between “number of hits” and any other markers tested, including femoral and tibial cartilage thickness, or pain severity in the ipsilateral knee. Arguably, potentially useful new knee OA biomarkers would not show positive associations with current measures of severity, given that these have very limited utility. Our findings that “number of hits” showed good intra- and inter-session reproducibility and differed on average between KL1 and KL2 suggest that this measurement may have potential applications in population-based cohort studies and clinical trials by measuring aspects not captured by currently-available measures.

“Number of hits” was consistently higher in movement quadrants 1 and 4 across the range of knee OA severity, suggesting that these phases within ascending and descending movements may be particularly informative, not only for clinical measurement but also for investigating origins for acoustic signals. Furthermore, whilst different quadrants are not distinguished by discrete cut-off points, it is notable that quadrants 1 and 4 correspond closely to movement phases which have been found, using instrumented knee implants, to generate the highest average loading resultant forces within knees [27]. Whilst the physical and clinical characteristics of that study group may not be analogous to ours, these findings nevertheless raise the possibility that “number of hits” may be related to joint loading forces generated during specific phases of standing and sitting movements.

Our finding that “number of hits” differs on average between KL1 and KL 2 but is also influenced by body weight and by pain in the contralateral knee suggests that AE may provide a composite readout, determined by structural change together with factors relevant to joint loading. The association of “number of hits” with both body weight and contralateral knee pain might be linked to altered biomechanics during sit-stand-sit movements. It has been reported that whilst people of normal weight produce hip torques which are higher than knee torques during sit-stand-sit movements, strategies used by obese people produce knee torques which are higher than hip torques [28]. Potentially, this may lead to higher “number of hits” in knees of obese people during sit-stand-sit movements. Furthermore, people with advanced knee OA transmit up to 10% more load through their contralateral leg during sit-stand-sit movements [29, 30]. Increased loading in the contralateral limb during gait has also been documented, and an association with subsequent development of damage in contralateral joints has been suggested [31–34]. Biomechanical changes in gait offer a possible rationale for explaining associations not only between “number of hits” and severity of knee OA as reflected by KL scores, but also between “number of hits” and contralateral knee pain, since higher loading through the contralateral leg due to knee OA may be responsible for contralateral knee pain. Future work to measure simultaneously “number of hits” and joint loading in knees during sit-stand-sit movements may provide a better understanding of the mechanisms involved, whilst deeper analysis of AE data according to defined localized anatomical features, or by identifying the most strongly repeatable AE waveform features, may reveal acoustic waveforms characteristic of specific features of joint condition.

Further modification of methods and materials for sensor attachment may reduce variance between sessions, for example by improving standardisation of sensor attachment, or improving consistency in sensor anatomical location. Reduction of between-session variance would reduce numbers of measurements needed while maintaining scientific value and user convenience of the method. This would support further the rationale for including AE measurement in knee OA clinical trials. For drug development, in both Europe [35] and United States [36], regulators will consider claims for slowing or prevention of structural damage. EMA notes that to assess effects of structure-modifying drugs, it may be advantageous to select patients at high risk for progression. Both FDA and EMA note that neither MRI nor radiography are ideal for evaluating OA severity, and recognise the need to develop alternative technologies. Since AE merits further investigation as one such technology, we have modelled a power calculation for a notional clinical trial of an intervention for knee OA which measured “number of hits” as one of its outcomes. Based on findings of the study reported here, a phase 2 clinical trial in people with KL scores of 2 or more would require approximately 400 participants per group to provide the trial with 80% power to demonstrate a 50% reduction in “number of hits”. Whilst such calculations are notional currently, and the clinical significance of such changes requires further investigation, they nevertheless indicate the feasibility of designing clinical trials along these lines.

In summary, measurement of AE number of hits using a simple sit-stand-sit movement protocol offers a novel and convenient approach for assessing the integrity of interactions between joint components during weight bearing movement. The results of this study demonstrate the potential for this method to enable the identification of new candidate biomarkers, prior to their subsequent validation in large-scale studies and future applications in multicentre clinical trials of knee OA. Whilst the key determinants of acoustic signals are yet to be elucidated, current work exploring associations between specific AE waveform features and defined structural features may help provide insights into this important issue. Future work will determine whether AE measurements should focus on specific movement phases, refine the equipment to enhance its usability in clinic settings and assess the role of frequency ranges used to capture AE signals.

## Supporting information

S1 Text Knee MR imaging studies. (DOCX)

S2 Text Bone and cartilage segmentation. (DOCX)

S3 Text Data analysis: Evaluating initial candidate AE biomarkers. (DOCX)

S1 Table Reproducibility of AE measurements in a concurrent study involving 73 other participants, with machines calibrated at a narrower frequency range of 20 kHz to 80 kHz. (DOCX)

S2 Table Estimated difference between Number of hits in the KL1 group and the KL2, KL3 and KL4 groups. (DOCX)

S3 Table Results from the univariable regression analysis of number of hits on patient characteristics, clinical markers and cartilage thickness. (DOCX)

S1 Fig Within-session reproducibility of AE candidate biomarkers. (DOCX)

S2 Fig The four phases of the sit-stand-sit movement protocol. (DOCX)

## Abbreviations

OA: Osteoarthritis
AE: Acoustic emission
JAAS: Joint acoustic analysis system
RP: NHS research practitioner
DD: Developmental dataset
PCA: Principle Component Analysis
SD: standard deviation
WOMAC: Western Ontario and McMaster Universities Osteoarthritis Index
