# Supplementary material for: Use of acoustic emission to identify novel candidate biomarkers for knee osteoarthritis (OA)
Source: PLoS One. 2019 Oct 16;14(10):e0223711. doi: 10.1371/journal.pone.0223711 (PMC6795455; doi:10.1371/journal.pone.0223711)
Supplement: S1 Text — (DOCX) [file pone.0223711.s001.docx]

# Supporting Information

**S1 Text**

**Knee MR imaging studies**

29 participants volunteered to undergo MR imaging of the worst knee within 4 weeks of AE and clinical assessment. MR images were acquired with a 3.0T Philips Achieva-X using a Philips 16-element SENSE knee coil with the knee stabilised with sandbags. Weight-bearing was minimised during the preceding 30 minutes. Four image sets of the knee were acquired: Firstly, a 2D multislice coronal turbo spin-echo sequence without fat saturation (FoV=140x140x123; acquisition matrix=290x384; analysis matrix=384x384; slices=41x3mm; FA=90, 180; TE=29; TR=3850; ETL=7); Secondly, a 3D sagittal gradient-echo sequence with water excitation (FoV=140x140x112; acquisition matrix=301x384; analysis matrix=384x384; slices=160x0.7mm; FA=25; TE=5.6; TR=15); Thirdly, a 2D multislice sagittal turbo spin-echo sequence with fat saturation (FoV=160x160x111; acquisition matrix=448x310; analysis matrix=448x448; slices=37x3mm; FA=90, 180; TE=30; TR=3200; ETL=5); Fourthly, a 2D multislice sagittal turbo spin-echo sequence without fat saturation (FoV=140x140x123; acquisition matrix=290x384; analysis matrix=384x384; slices=123x1mm; FA=90, 180; TE=29; TR=3850; ETL=7).
