# Supplementary material for: Use of acoustic emission to identify novel candidate biomarkers for knee osteoarthritis (OA)
Source: PLoS One. 2019 Oct 16;14(10):e0223711. doi: 10.1371/journal.pone.0223711 (PMC6795455; doi:10.1371/journal.pone.0223711)
Supplement: S2 Text — (DOCX) [file pone.0223711.s002.docx]

# Supporting Information

**S2 Text**

**Bone and cartilage segmentation**

Sagittal fat-saturated MR images were acquired using a Philips 3D WATSf sequence; flip angle 25°, TE 5.6 ms, TR 15 ms, pixel size 0.36 x 0.36mm, slice thickness 0.7mm. Bone and cartilage were segmented by an experienced manual segmenter, using a semi-automated livewire algorithm (Endpoint segmentation software, Imorphics, Manchester, UK). A set of contours, one for each slice of the MR image 3D surfaces were then generated from the stack of contours using a marching- cubes algorithm, followed by quadratic smoothing of the resultant surfaces. Femur, tibia and patella bone surfaces were automatically segmented using active appearance models (AAMs), built from an independent training set of 96 knee MRIs using the Siemens DESS-we sequence . This training set was selected to contain approximately equal numbers for each KL grade. The AAMs were applied to the same 3D WATSf sequences. During auto-segmentation with AAMS, each bone was fitted with a dense set of correspondence points (landmarks), for the femur this set of points is >32,000 with each point separated by around 1mm.

At this stage, each bone surface exists in 2 forms (i) the manual bone surface, and (ii) the AAM bone surface. Correspondence points from the AAM bone surface were transferred onto the manual bone surface by projecting normal from each correspondence point so as to intersect with the manual bone surface. This produced an accurate manual segmented bone surface, fitted with a set of anatomical landmarks; these were used for comparisons across the population, and for alignment and rotation of bones. Cartilage thickness measures were taken using specified correspondence points on bone surfaces, and measuring distances to the outside of manually-segmented cartilage surfaces, normal to that surface [S2 Text, 1]

**References**

S2 Text, 1. Eckstein F, Ateshian G, Burgkart R, Burstein D, Cicuttini F, Dardzinski B et al Proposal for a nomenclature for magnetic resonance imaging based measures of articular cartilage in osteoarthritis. Osteoarthritis Cartilage 2006; 14(10): 974-83 Epub 2006 May 26
