# Supplementary material for: Use of acoustic emission to identify novel candidate biomarkers for knee osteoarthritis (OA)
Source: PLoS One. 2019 Oct 16;14(10):e0223711. doi: 10.1371/journal.pone.0223711 (PMC6795455; doi:10.1371/journal.pone.0223711)
Supplement: S3 Text — (DOCX) [file pone.0223711.s003.docx]

# Supporting Information

**S3 Text**

**Data analysis: Evaluating initial candidate AE biomarkers**

The four AE candidate biomarkers were evaluated in two ways

- 1. With respect to associations with participant characteristics and disease specific markers
  2. With respect to reproducibility.

The general modelling framework was as follows. Let *Y* denote a candidate biomarker. We modelled any obtained value by

| $y_{ijtr}=x_{i}^{'}\alpha+U_{i}+V_{j}+W_{it}+Z_{ijtr}$ | (1) |
| --- | --- |

where *i* is the participant, *j* is the practitioner taking the measurement, *t* is the time unit and *r* is the repeat. 𝑦𝑖𝑗𝑡𝑟 is the candidate biomarker value obtained from participant *i* by practitioner *j* during repeat *r* in time unit *t*. *xi* is a vector of explanatory variables for participant *i*, including the JAAS machine used for the patient in question, and *α* the corresponding vector of regression coefficients to be estimated. *Ui* is a participant-specific random effect that accounts for correlation between repeated measurements within the same individual after adjustment for the covariates *xi*. *Vj* is a random effect capturing the variability between practitioners, *Wit* captures the variability between time units within participants, and *Zijtr* is a residual error term. All random effect terms were assumed to be Normally distributed with zero-mean.

1. Associations with participant characteristics and disease specific markers

To assess the associations of AE candidate biomarkers with other markers and patient characteristics a special case of (1) was fitted to data collected during the first session:

$$y_{ir}=x_{i}^{'}\alpha+U_{i}+Z_{ir}$$

A multiple regression model was developed using forward selection of the covariates *x*i based on the likelihood ratio test with a cut-off for significance of p<0.1. The variables assessed as potential covariates are age, sex, weight, BMI, KL score, WOMAC pain

score, WOMAC stiffness score, WOMAC function score, visual analogue scale (VAS) pain score, presence/absence of pain in the other knee, JAAS machine and measurement batch (measurements were separated into 2 batches due to some adjustment to the JAAS machines during the study).

The model was first developed on 68 participants who had complete data and then refitted using data from 76 participants for whom complete data on the covariates used in the final model were available.

1. Reproducibility

Reproducibility was assessed using data from the 45 individuals who consented to take part in the reproducibility studies.

To assess the variability between machines, between practitioners and between visits within patients within practitioners, and to account for the variability introduced by the application of the sensors, which is not necessarily a systematic difference between practitioners, two separate linear mixed effects models of type (1) were fitted; a longitudinal model and a ‘day one’ model.

The time unit *t* was ‘day’ in the longitudinal model and 'session' in the ‘day one’ model. A ‘session’ is a time unit during which the sensors are not removed and re-applied.

16

Differences in biomarker values between the JAAS machines were estimated by the regression coefficients for machines two and three relative to machine one.

The standard deviations of the random effect terms were estimated and give a measure of the variability of the biomarkers attributable to these individual components.

Two versions of each model were considered, one in which we only adjusted for JAAS machine, the other in which we adjusted for patient characteristics found to be associated with the biomarker values in the previous analysis, i.e. weight, age, sex, VAS pain score in the indicator knee rated by the individual in the same week as biomarker was measured, KL score of the indicator knee, presence/absence of pain in the other knee. The models were fitted to each of the three AE biomarkers and the number of hits separately.

Before analysis, a Box-Cox transformation [S3 Text, 1] with parameter 0.2 was performed on the candidate AE biomarker 3 to give a more symmetric distribution. Also, age, BMI and weight were centred at their mean values 69, 30 and 84, respectively, to ease interpretation of the estimated intercept of the regression model. To assess the association between KL scores and biomarker values, forward difference contrasts for the KL scores were used.

All analyses were performed using the statistical computing environment R and the `lme4' package. Parameters in the final models were estimated using restricted maximum likelihood. The confidence intervals provided were calculated using the ‘profile’ option in the ‘methods’ argument of the lme4 function *confint*.

**References**

[S3 Text, 1] Box, G. E. P. and Cox, D. R. An analysis of transformations. Journal of the Royal Statistical Society, 1964; Series B, 26: 211-252.
