# Supplementary material for: Use of acoustic emission to identify novel candidate biomarkers for knee osteoarthritis (OA)
Source: PLoS One. 2019 Oct 16;14(10):e0223711. doi: 10.1371/journal.pone.0223711 (PMC6795455; doi:10.1371/journal.pone.0223711)
Supplement: S1 Table — (DOCX) [file pone.0223711.s004.docx]

# Supporting Information

**S1 Table**

**Reproducibility of AE measurements in a concurrent study involving 73 other participants, with machines calibrated at a narrower frequency range of 20 kHz to 80 kHz**

Table S1: Part A: Point estimates and 95% confidence intervals for the standard deviations of the random effect terms and the regression coefficients of the JAAS machine relative to JAAS 1 in the longitudinal model of the ‘Number of hits’. (LCL: lower confidence limit; UCL: upper confidence limit)

Table S1: Part B: Point estimates and 95% confidence intervals for the standard deviations of the random effect terms and the regression coefficients of the JAAS machine relative to JAAS 1 in the ‘day 1’ model of the ‘Number of hits’. (LCL: lower confidence limit; UCL: upper confidence limit)

| **Parameter** | **Model without covariate adjustment** | | | **Model with covariate adjustment** | | |
| --- | --- | --- | --- | --- | --- | --- |
|  | **Point Estimate** | **95% LCL** | **95% UCL** | **Point Estimate** | **95% LCL** | **95% UCL** |
| **A** | | | | | | |
| Day in patient variability | 19.78 | 17.84 | 21.94 | 18.91 | 16.98 | 21.03 |
| Patient variability | 33.11 | 27.29 | 39.84 | 28.57 | 21.42 | 32.00 |
| RP variability | 8.24 | 0.00 | 20.20 | 3.72 | 0.00 | 14.28 |
| Residual variability | 12.97 | 12.06 | 14.01 | 10.98 | 10.15 | 11.93 |
| JAAS 2 | 6.16 | -14.66 | 28.31 | 15.78 | -2.92 | 34.49 |
| JAAS 3 | 3.55 | -17.82 | 24.08 | -1.95 | -20.79 | 17.52 |
| **B** | | | | | | |
| Session in patient variability | 19.97 | 17.34 | 23.21 | 18.97 | 15.98 | 23.20 |
| Patient variability | 36.52 | 29.84 | 43.26 | 33.32 | 24.27 | 37.02 |
| RP variability | 9.15 | 0.00 | 18.65 | 13.57 | 0.00 | 30.65 |
| Residual variability | 10.53 | 9.59 | 11.62 | 9.71 | 8.79 | 10.81 |
| JAAS 2 | 6.42 | -14.76 | 27.43 | 7.04 | -14.03 | 27.81 |
| JAAS 3 | 4.78 | -18.05 | 27.42 | -4.95 | -28.74 | 18.09 |
