# Supplementary material for: Use of acoustic emission to identify novel candidate biomarkers for knee osteoarthritis (OA)
Source: PLoS One. 2019 Oct 16;14(10):e0223711. doi: 10.1371/journal.pone.0223711 (PMC6795455; doi:10.1371/journal.pone.0223711)
Supplement: S2 Table — (DOCX) [file pone.0223711.s005.docx]

# Supporting Information

**S2 Table**

**Estimated difference between Number of hits in the KL1 group and the KL2, KL3 and KL4 groups.** We re-fitted our final model, however instead of using forward-difference contrasts for the KL groups, we estimated the difference of the number of hits in the KL1 group compared to KL2, KL3 and KL4 groups using dummy coding. We did not find significant difference between the number of hits measured in the KL1 group compared to the KL3 and KL4 groups.

|  | Point Estimate | 95% LCL | 95% UCL |
| --- | --- | --- | --- |
| Intercept | 119.68 | 62.32 | 177.03 |
| KL2 vs KL1 | 81.77 | 20.13 | 143.41 |
| KL3 vs KL1 | 59.23 | -2.12 | 120.58 |
| KL4 vs KL1 | 20.84 | -58.39 | 100.07 |
| Weight | 2.06 | 0.76 | 3.37 |
| Pain in contralateral knee | 57.05 | 14.13 | 99.97 |
| Standard deviation of participant specific random effect | 93.17 | 76.58 | 105.98 |
| Standard deviation of residual error | 22.51 | 19.39 | 26.57 |
