# Supplementary material for: Use of acoustic emission to identify novel candidate biomarkers for knee osteoarthritis (OA)
Source: PLoS One. 2019 Oct 16;14(10):e0223711. doi: 10.1371/journal.pone.0223711 (PMC6795455; doi:10.1371/journal.pone.0223711)
Supplement: S3 Table — (DOCX) [file pone.0223711.s006.docx]

# Supporting Information

**S3 Table**

**Results from the univariable regression analysis of number of hits on patient characteristics, clinical markers and cartilage thickness**

Table S3: Part A: Deviance and p-values from likelihood ratio test resulting from adding the patient characteristics and clinical markers variables one at a time to the random intercept model for number of hits.

Table S3: Part B: Deviance and p-values from likelihood ratio test resulting from adding MRI summary statistics one at a time to the random intercept model for number of hits.

| **Covariate** | **deviance** | **df** | **p-value** |
| --- | --- | --- | --- |
| **A** | | | |
| WOMAC pain score | 0.47 | 1 | 0.49 |
| WOMAC stiffness score | 0.25 | 1 | 0.62 |
| WOMAC function score | 0.32 | 1 | 0.57 |
| Age | 0.57 | 1 | 0.45 |
| Male | 1.37 | 1 | 0.24 |
| Pain in contralateral knee | 4.08 | 1 | 0.04 |
| BMI | 5.84 | 1 | 0.02 |
| Weight | 10.58 | 1 | <0.01 |
| VAS score | 0.46 | 1 | 0.50 |
| **B** | | | |
| cartilage thickness on central section on medial femur | 0.26 | 1 | 0.61 |
| cartilage thickness on central section on medial tibia | 0.02 | 1 | 0.90 |
