# Supplementary material for: Use of acoustic emission to identify novel candidate biomarkers for knee osteoarthritis (OA)
Source: PLoS One. 2019 Oct 16;14(10):e0223711. doi: 10.1371/journal.pone.0223711 (PMC6795455; doi:10.1371/journal.pone.0223711)

# Supporting Information

**S1 Figure**

**Within-session reproducibility of AE candidate biomarkers.** Each graph shows the biomarker value obtained from the first set of sit-stand-sit movements (x-axis) plotted against the biomarker value obtained from the repeat set of movements (y-axis). This plot includes the outlier in the Number of hits which was omitted from Figure 3 in the main manuscript.


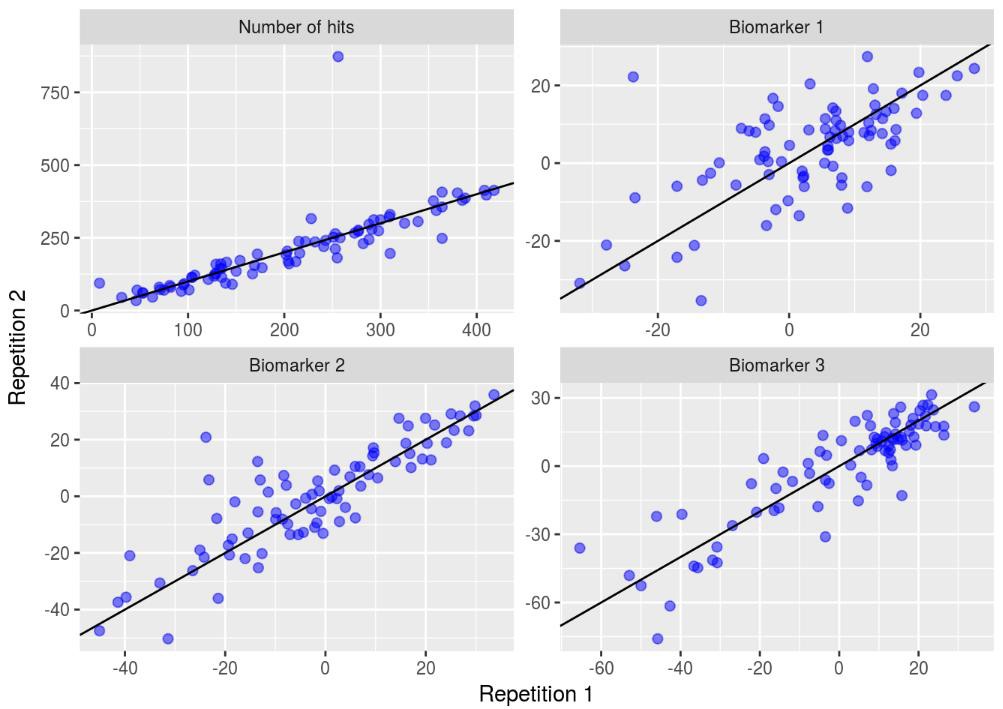

Supplement: S1 Fig — (DOCX) [file pone.0223711.s007.docx]
