# Supplementary material for: Use of acoustic emission to identify novel candidate biomarkers for knee osteoarthritis (OA)
Source: PLoS One. 2019 Oct 16;14(10):e0223711. doi: 10.1371/journal.pone.0223711 (PMC6795455; doi:10.1371/journal.pone.0223711)

# Supporting Information

**S2 Figure**

**The four phases of the sit-stand-sit movement protocol** The phases are defined as follows: Ascending-Acceleration phase (AA): from the start of the movement at the sitting position to the occurrence of the peak angular velocity; Ascending-Deceleration phase (AD): from the peak angular velocity in ascending to the occurrence of the angle at the standing position; Descending-Acceleration phase (DA): from the occurrence of the angle at the standing position to the occurrence of the peak angular velocity in descending; and Descending-Deceleration phase (DD): from the peak angular velocity to the stop of the movement at the sitting position. Angular velocity greater than 0.1 degrees per second was used to define the start of the movement and angular velocity less than 0.1 degrees was used to define the end of the movement.


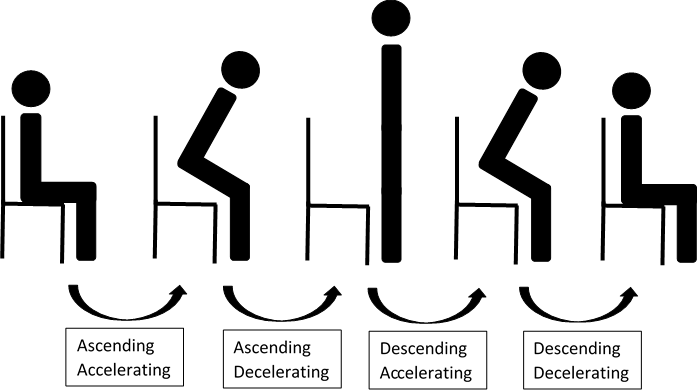

Supplement: S2 Fig — (DOCX) [file pone.0223711.s008.docx]
